# Rapid, easy, and cheap randomization: prospective evaluation in a study cohort

**DOI:** 10.1186/1745-6215-13-90

**Published:** 2012-06-22

**Authors:** Melissa J Parker, Asmaa Manan, Mark Duffett

**Affiliations:** 1 Division of Critical Care, Department of Pediatrics, McMaster Children’s Hospital and McMaster University, 1200 Main St W. Room 3A-80, Hamilton, ON L8N 3Z5, Canada; 2 Division of Emergency Medicine, Department of Pediatrics, The Hospital for Sick Children and The University of Toronto, Toronto, Canada; 3 Undergraduate Student, McMaster University, 1200 Main St W. Room 3A, Hamilton, ON, L8N 3Z5, Canada; 4 Division of Critical Care, Department of Pediatrics, McMaster Children’s Hospital and McMaster University, 1200 Main St W. Room 1E1A, Hamilton, ON, L8N 3Z5, Canada

**Keywords:** Randomization, Allocation, Clinical trials, Computers, Handheld

## Abstract

**Background:**

When planning a randomized controlled trial (RCT), investigators must select randomization and allocation procedures based upon a variety of factors. While third party randomization is cited as being among the most desirable randomization processes, many third party randomization procedures are neither feasible nor cost-effective for small RCTs, including pilot RCTs. In this study we present our experience with a third party randomization and allocation procedure that utilizes current technology to achieve randomization in a rapid, reliable, and cost-effective manner.

**Methods:**

This method was developed by the investigators for use in a small 48-participant parallel group RCT with four study arms. As a nested study, the reliability of this randomization procedure was prospectively evaluated in this cohort. The primary outcome of this nested study was the proportion of subjects for whom allocation information was obtained by the Research Assistant within 15 min of the initial participant randomization request. A secondary outcome was the average time for communicating participant group assignment back to the Research Assistant. Descriptive information regarding any failed attempts at participant randomization as well as costs attributable to use of this method were also recorded. Statistical analyses included the calculation of simple proportions and descriptive statistics.

**Results:**

Forty-eight participants were successfully randomized and group allocation instruction was received for 46 (96%) within 15 min of the Research Assistant placing the initial randomization request. Time elapsed in minutes until receipt of participant allocation instruction was Mean (SD) 3.1 +/− 3.6; Median (IQR) 2 (2,3); Range (1–20) for the entire cohort of 48. For the two participants for whom group allocation information was not received by the Research Assistant within the 15-min pass threshold, this information was obtained following a second request at 18 and 20 min, respectively. The method described here produced an email audit trail, which proved useful to the primary study.

**Conclusions:**

We report a method of third party randomization that uses current technology to operationalize randomization and allocation in a rapid, easy, and cost-effective manner. Other investigators may find this method useful, particularly for small RCTs, including pilot RCTs, on a tight budget.

## Background

Well-conducted and adequately powered randomized controlled trials (RCTs) represent the highest level of evidence upon which clinical practice recommendations are based [1,2]. This is because participant randomization helps to balance known and unknown confounding variables between experimental and control groups, isolating the effects of a given intervention on outcome [3]. While junior researchers may have a theoretical understanding of RCT methodology, there are pragmatic issues related to randomization procedures with which they may have little experience.

As a new researcher, one may be surprised to learn the cost associated with tamper-proof randomization processes. For example, one web-based randomization service, http://randomize.net, charges $2,500 (Canadian) for randomization services for a single trial regardless of size. Similarly, the rate for randomization services provided by a statistician at our university (McMaster University) is $3,000 (Canadian) annually per trial, based on a recently obtained quote (September, 2011). Fees for these services generally include preparation of the randomization sequence, including concealment, stratification, and blocking if required, for single or multiple centers, and a mechanism by which allocation can be accomplished.

While less expensive methods of operationalizing randomization exist, many of these methods have the potential to introduce bias through ineffective concealment of the allocation sequence. Pseudorandomization or treatment allocation according to factors such as the day of the week, odd/even calendar dates, or using digits of the hospital ID number, provides no allocation concealment. Randomization using envelopes to assign participants to a treatment group is another simple strategy that attempts to conceal the allocation sequence, however concealment may be jeopardized through a variety of means [4,5]. Unconcealed allocation may lead to between-group differences apart from the intervention of interest, and has the potential to influence outcome, including over- or under-estimation treatment effect [4,6,7].

Developing a randomization sequence in and of itself is not difficult. Random numbers tables, random number generators, or other computer programs may be used for this purpose [8]. From a practical perspective, the greater issues relate to: (1) having the randomization sequence generated independently from and inaccessible to the research team; (2) developing a reliable means by which study team members can request participant randomization once consent has been obtained; and (3) ensuring timely transmission of treatment allocation information to the study team so that study interventions can be initiated in a timely manner. In this paper, we describe and relate our single-center experience with a simple and cost-effective randomization procedure that preserves allocation concealment. We also discuss practical considerations relevant to investigators in selecting a randomization and allocation method for an RCT.

## Methods

We developed and implemented a third party randomization procedure for use in a Research Ethics Board approved, 48 participant parallel group RCT. The trial, conducted at McMaster University Medical Centre (Hamilton, Ontario) from October to December 2011, included consenting healthcare provider participants who were asked to perform a resuscitation intervention on a low-fidelity simulator according to random assignment to one of four study arms. The trial was conducted in accordance with the Tri-council Policy Statement. [9] In a nested study, we prospectively evaluated the reliability of our randomization procedures.

In evaluating our randomization procedures specifically, the primary outcome of interest was the proportion of participants for whom allocation information was obtained by the Research Assistant within 15 min of the initial request time. We selected a 15-min time interval as the threshold because trials involving resuscitation interventions typically have brief windows during which eligibility assessments must be made, consent procured, and randomization procedures conducted to enable participation. For other trials, timely randomization and receipt of allocation information remains important, although less time sensitive. A secondary outcome of this nested study was to determine the average time (mean, sd) for communicating group assignment back to the requesting individual. Descriptive information regarding the frequency and circumstances surrounding failed attempts at randomization and costs attributable to our randomization procedures were also recorded. The sample size of the study cohort was dictated by the sample size requirements of the primary study.

### Random sequence generation

A clinician researcher not otherwise involved in the primary study (MD) functioned as a third party randomization coordinator for this trial. In this role, MD prepared a randomization schedule using the freely accessible tools available at http://www.randomization.com/. The sequence generated included randomly varying blocks of four and eight participants (six and three blocks, respectively) [8,10]. Seed 2276 may be used to exactly reproduce the randomization schedule from our trial at http://www.randomization.com/, with knowledge of the size and number of blocks and the group labels used (10 mL, 20 mL, 30 mL, 60 mL). Please note that block details and labels must be entered in the same order and orientation, and so the Randomization Coordinator should keep a record of this. Block randomization was used to mitigate the effect of any potential differences in participants or trial conduct over time and to reduce the risk of unequal group sizes in the event that the planned sample size was not attained [3,4]. To allow for flexible and convenient access to the randomization schedule from anywhere, MD converted it to a spreadsheet accessible via Google Docs, copyright of Google Inc. (Google Inc., Mountain View, CA, USA) using the web browser of a computer or smartphone. He also carried a paper copy of the schedule on his person as a backup in the event that the web-based schedule could not be accessed due to unforeseen circumstances.

### Participant randomization and allocation

Once each participant arrived at the testing location and provided consent to participate in the study, the Research Assistant (AM) sent the Randomization Coordinator a text message containing the participant’s study ID number. Upon receipt of the text message request, the Randomization Coordinator consulted the randomization schedule using the web browser of his smartphone or computer. He then sent an electronic mail (email) containing the participant’s study ID number and group allocation to the study email address. If the Research Assistant did not receive the allocation information within 15 min of the request being placed, she contacted the Randomization Coordinator by telephone to secure this information.

The Research Assistant used an iPad 2 tablet device (32 GB with Wi-Fi, Apple model A1395), trademark of Apple Inc. (Apple Inc., Cupertino, CA, USA), for all study procedures. The study email account was accessed via the iPad using the Mail program (version 4.5, Apple Inc.). The push feature of the iPad resulted in incoming messages to the study email account being automatically uploaded to the Mail program in real time. The Mail program alerted the Research Assistant to receipt of the participant group assignment email by two mechanisms: (1) an auditory alert; and (2) the Mail icon on the iPad desktop illustrating a new pending message. Randomization time was predefined as the amount of time that elapsed between the Research Assistant sending the first text message requesting participant randomization and subsequently receiving participant group allocation information.

We chose to use a combination of text messaging and emails as part of our randomization and allocation procedures because these are fast, common, and convenient forms of communication used in the present day and age. In our study setting, text messaging relied upon the availability of cellular network coverage while email functionality relied upon adequate Wi-Fi (wireless Internet) signal to the iPad. Both forms of communication permitted mobile messaging. From a confidentiality perspective, text messages were deleted following receipt and the email account was only accessible to the investigators of the primary trial through use of a secure password. The rationale for the use of email to transmit participant group assignment information was because this resulted in the creation of an audit trail for both the investigators and the Randomization Coordinator.

## Results

Forty-eight participants were successfully randomized. Of these, group allocation instruction was received for 46 (96%) within 15 min of the Research Assistant placing the randomization request. In one instance, the Research Assistant contacted the Randomization Coordinator prior to reaching the 15-min threshold. As such, pass/fail data regarding the performance of our method (per protocol) is unavailable for this participant. Allocation information was not received within 15 min for two of 48 participants (4%). In the first instance, there was a delay in receiving the allocation information via email due to a loss of Wi-Fi signal to the iPad. In the second instance, the Randomization Coordinator had moved away from his smart phone and missed the initial notification. The median time for receipt of participant allocation instruction from the time of the initial randomization request for our cohort was 2 min. Detailed information regarding participant randomization time is presented in Table 1.

**Table 1:** Time to receipt of participant allocation information and success rate per protocol

**Randomization per protocol within 15 min threshold**	***n***	**Time elapsed in minutes from randomization request to receipt of allocation information**	
Pass	45	Mean (SD)	2.3 (1)
		Median (IQR)	2 (2,3)
		Range	1-5
Fail	2	Mean (SD)	19 (1.4)
		Median	19
		Range	18-20
Unknown	1	Time	7
All	48	Mean (SD)	3.1 (3.6)
		Median (IQR)	2 (2, 3)
		Range	1-20

*IQR*, interquartile range; *SD*, standard deviation.

The total cost of our randomization procedure was $250 (Canadian). This included creation of the randomization sequence and a nominal fee of $5 per participant randomized. No additional equipment was purchased in relation to the randomization procedures used. Wireless Internet access was freely available to the research team at our institution and research staff used their personal smart phones and incurred no additional charges in this study.

## Discussion

Principal investigators must consider a variety of factors when establishing randomization procedures for a clinical trial. In weighing the pros and cons of different methods, important considerations include the strength of allocation concealment, cost (especially for pilot trials), timely receipt of allocation information, and confidentiality (Table 2). The use of an independent third party to perform central randomization is considered among the most desirable methods to effectively conceal allocation. [11] In this report we describe a single-center experience with a simple and inexpensive randomization method that preserves allocation concealment. This strategy involves an independent Randomization Coordinator who generated a confidential randomization sequence that was inaccessible to study investigators and potential trial participants.

**Table 2:** Considerations in selecting a randomization and allocation strategy

**Method**	**Best setting**	**Cost**	**Advantages**	**Disadvantages**
Web-based randomization	Multisite	High	- High reliability 24 h per day	-Dependent on Internet functionality
			- Capacity to handle large volume of randomization requests	- Requires access to a device (usually a computer) to access the Internet
			- Electronic audit trail created	- Requires familiarity with process
				- High cost
Telephone accessible central coordinating site	Multisite	High	- High reliability 24 h per day	- Dependent on access to a working telephone
			- Additional resources associated with a staffed coordinating site	- Requires staffing of central coordinating site
			- Audit trail created	- High cost
Third party prepared sealed packages of identical appearance	Single site, multisite possible	Moderate	- High reliability 24 h per day	- Research Assistant or enrolling clinician must know where to find packages consistently
			- Little time required to obtain participant allocation when implemented optimally	- Vulnerable to breach of allocation sequence
				- Important to ensure treatment assignment cannot be discerned from package features (size, weight)
				- Preparation time/cost
Text message/email method described in this paper	Single site, multisite not evaluated	Low	- High reliability demonstrated in a small cohort	- Requires both Research Assistant and Randomization Coordinator to have functional smart phone on person with cellular network connectivity
			- Allocation details received quickly	- Requires Research Assistant to have functional device that can access study email account via Wi-Fi or cellular network
			- Email audit trail created	- Method performance during unsociable hours not yet assessed
			- Low cost	
Sequentially numbered, opaque sealed envelopes	Single site	Low	- High reliability	- Research Assistant must be able to access envelopes consistently
			- Allocation details received quickly	- Vulnerable to breach of allocation sequence
			- Audit trail created (allocation paper)	- Participant personal information transferred to allocation paper
			- Low cost	
Coin toss	Single site	Low	- Easily accessible	- Only simple randomization (no blocking)
			- Low cost	- No audit trail
				- Vulnerable to corruption

The actual costs related to use of this method will differ between trials based upon the resources available, the number of participants, trial complexity, and time constraints. The $250 (Canadian) randomization cost for the RCT was deemed reasonable as each randomization request required approximately 30 s of the Randomization Coordinator’s time to respond to it. Use of a smart phone to receive randomization requests, consult the randomization schedule, and to accomplish the tasks necessary to generate the allocation email also meant that this work had minimal impact on his other activities. For our relatively small randomized trial, use of the randomization method described herein resulted in a significant cost savings in comparison to other methods with robust allocation concealment: including randomization by a statistician, web-based randomization, and other central randomization schemes. In our case, costs related to use of one of these more established randomization techniques would have been prohibitive and consumed more than 50% of our total study budget.

While other methods such as use of sequentially numbered, opaque sealed envelopes represent a suitable alternative randomization strategy when third party randomization is not feasible [12,13], this method is vulnerable to corruption. [4] To properly operationalize the opaque envelope method, one must also account for the costs related to purchase of envelopes, carbon paper, cardboard, or foil to ensure opacity, and the time required to properly package all materials. This alternative method also has its own special requirements, as it has been recommended that the name and details of a participant should be written on the envelope prior to opening in a manner that achieves irreversible transfer of this data onto the allocation paper. [12] With increasingly stringent privacy laws, such transfer of patient identifiers may become increasingly problematic. Despite precautions, the opaque envelope method also remains vulnerable to envelopes being accessed out of sequence, other breaches of allocation concealment, or accessibility problems such as envelopes being locked away in a distant office.

Our method was simple, easy to implement, and effective, with 96% of participant randomizations occurring within our 15-min target. In the two instances where allocation information was not received within the 15-min threshold, the causes are known and not insurmountable. The first case was a technical problem due to loss of Wi-Fi signal resulting in a delay in transmission of the allocation information to the study email account. This type of problem can easily be prevented by use of a mobile device (smart phone, iPad) with Wi-Fi and cellular network connectivity. Researchers should consider the reliability of the cellular network in their area and the consequences of any period(s) of disruption. A stationary device with a hardwired network connection may also be feasible in some trials. In the second instance, the Randomization Coordinator had moved away from his smart phone and missed the initial notification, demonstrating that our method is still vulnerable to human factors. Based on our experiences with this small cohort, we recommend planning an alternative method of communication as a back-up method by which participant randomization can be achieved should the primary method fail. Options that may be appropriate include a standard pager, a second telephone number, or involvement of an alternative person with access to the randomization schedule who is not otherwise involved in the study. We also recommend that study staff should confirm the receipt of the randomization information within a pre-specified time.

Researchers considering the use of similar methods should also ensure adequate study procedures to prevent and detect any randomization errors. In our case, creation of an email audit trail proved useful following completion of the RCT as it was discovered that the same allocation instruction had been sent for two consecutive participants. We recommend that each participant’s study number be included with the group allocation and that this be confirmed prior to the commencement of the study procedure.

In addition to considering the practical aspects of each method, researchers must also address the security of study information and the privacy and confidentiality of participants and their personal information. To ensure that the allocation sequence remains concealed, the Randomization Coordinator should not be otherwise involved in the study. Beyond this, the lock function should be used to limit access to any portable electronic devices and strong passwords should be employed to restrict access to the randomization schedule. Any paper back-up copies of the allocation sequence must be securely stored. The most straightforward approach to protecting privacy and confidentiality is to avoid sending personal identifiers via the Internet and/or cellular networks and to communicate using only the participant study number. The addition of participant initials or a portion of an ID number are options to make the allocation process more robust, but use must comply with the relevant regulations and guidelines. Researchers must also carefully consider any stratification variables, which may include personal health information. Technical solutions for the transmission of confidential information are also available, but evaluation and implementation may be too complex for a smaller trial. Researchers should consult the applicable standards in their jurisdiction on encryption, transmission, and the methods and location of data storage. Using existing hospital or other research institution networks may be a simpler option, as free or low-cost commercial services may not meet the required standards.

As this study included a small number of participants, it is possible that this method is neither feasible nor cost effective for use in larger trials. High volume trials with frequent and/or numerous randomization requests may require multiple Randomization Coordinators to share the responsibility, particularly for trials operating 24 h per day. Using a per participant randomization fee for larger trials may also result in costs exceeding the flat fee charged by web-based randomization services. Researchers should also consider the sustainability of this method for trials of long duration. In addition to the features of the specific trial, costs will also depend on the other resources available to the Principal Investigator. Because our trial was conducted at a single site, we acknowledge that additional costs may also be incurred in the setting of a multicenter trial. The method that we used involved text messaging the Randomization Coordinator, and as such charges related to text messaging or long distance paging may also need to be considered. Further adaptations to the method described here may circumvent some of these issues that have the potential to generate additional costs.

## Conclusions

In summary, we report a method of third party randomization that uses current technology to operationalize randomization in a rapid, easy, and cost effective manner. This method or adaptations of it may be appropriate for use in other RCTs, including multicenter trials, where it is determined to be a feasible and cost-effective alternative to conventional randomization strategies. Suggestions are provided for those considering use of an approach similar to that described here.

## Abbreviations

Email: Electronic mail; RCT: Randomized controlled Trial; Wi-Fi: Wireless Internet.

## Competing interests

MP - MP is an Assistant Professor of Pediatrics, McMaster University, and a staff physician at McMaster Children’s Hospital. MP has received research start-up funding from McMaster and some of these funds may be used if required to cover publication costs in relation to this article. McMaster University and McMaster Children’s Hospital may benefit in reputation from the publication of this article. AM - No competing interests to declare. MD - No competing interests to declare.

## Authors’ contributions

MP conceived of the method by which participant randomization and allocation could be accomplished quickly and cheaply using current technology and had the idea to study this prospectively. MP developed the protocol, entered the data, performed the statistical analyses, and wrote the initial draft of the manuscript. AM was responsible for data collection and contributed to the final version of the manuscript. MD acted as the Randomization Coordinator and contributed to the final version of the manuscript. All authors read and approved the final manuscript.

## Authors’ information

MP is an Assistant Professor of Pediatrics at McMaster University and an Adjunct Clinical Assistant Professor of Pediatrics at the University of Toronto. Her research interests relate to pediatric resuscitation interventions, including the logistical and methodological challenges of doing research in this area. AM is currently an MSc student in Biomedical Engineering at McMaster University. MD is an Assistant Professor (part time) in the Department of Pediatrics and a PhD student in Health Research Methodology at McMaster University.
